# Anti-infective Efficacy of Mechanical Debridement with Adjunctive Modalities on Clinical and Cytokine Parameters in Treatment of Chronic Periodontitis: Randomized Controlled Clinical Trial

**DOI:** 10.1055/s-0043-1772567

**Published:** 2023-10-17

**Authors:** Anjale Rajagopal, Jothi Varghese, Vinutha Bhat, Vibha Acharya, Vinayak Kamath

**Affiliations:** 1 Department of Periodontology, Manipal College of Dental Sciences, Manipal Academy of Higher Education, Manipal, Karnataka, India; 2 Department of Biochemistry, Kasturba Medical College, Manipal Academy of Higher Education, Manipal, Karnataka, India; 3 Department of Public Health Dentistry, Goa Dental College and Hospital, Bambolim, Goa, India

**Keywords:** lasers, tissue desiccant, inflammatory mediators, mechanical debridement, chronic periodontitis

## Abstract

**Objectives** Periodontal debridement involves conventional scaling and root planing (SRP) along with variant forms of adjunctive therapies. In the present clinical trial, we investigated if the adjunctive use of HybenX gel or diode laser along with SRP could provide a favorable outcome for the treatment of chronic periodontitis.

**Materials and Methods** The present study involved 60 subjects diagnosed with chronic periodontitis who were randomly assigned as test groups (laser or HybenX) or control group (SRP alone). The primary outcomes of the clinical trial were pocket probing depth (PPD) and clinical attachment level (CAL), which was evaluated at baseline and at third-month time interval. Additionally, secondary outcomes included estimation of reduction in inflammatory mediators interleukin 1β (IL-1β) and tumor necrosis factor α (TNF-α) in gingival crevicular fluid using enzyme-linked immunosorbent assay at baseline and third-month intervals.

**Statistical Analysis** Normality determination was checked using Shapiro–Wilk test. Since the data was not normally distributed, nonparametric tests were applied. The comparison of clinical parameters between the groups was analyzed with Kruskal–Wallis test. Wilcoxon sign rank test was used to compare the pairwise comparison of clinical parameters among the groups from baseline to third-month follow-up. The inflammatory mediators at various time points were compared using a One-way analysis of variance test, and the inflammatory mediators in each study group were compared using a paired *t* -test.

**Results** Both the test groups demonstrated a decrease in PPD and CAL. However, the HybenX group exhibited statistically significant reduction at the end of the third-month study interval compared to the laser group and SRP alone. Further, the secondary outcome IL-1β and TNF-α analysis exhibited statistically significant reduction in all the groups posttherapy.

**Conclusion** The adjunctive application of HybenX gel yielded an advantage compared to laser and SRP for the treatment of chronic periodontitis.

**Clinical Relevance** Adjunctive use of the oral tissue desiccant (HybenX gel) combined with SRP improved the periodontal pocket disinfection process and enhanced tissue healing devoid of adverse effects.

## Introduction

Periodontal root debridement is an integral constituent of both nonsurgical and surgical pocket therapy that facilitates predictable outcome of achieving gain in periodontal attachment on formerly infected root surfaces. 1 The strategic optimal management for periodontitis involves scaling and root planing (SRP) which has shown favorable outcome. 2 However, this factual therapy does not provide complete elimination of pathogenic biofilm, particularly in deep subgingival environment and inaccessible areas resulting in reinitiation of inflammatory process.

Periodontitis has been described as a host-mediated disruption of microbial homeostasis and it is well understood that by controlling inflammation either through conventional mechanical therapy or pharmacological adjuncts, there is a possibility to limit the infection. 3 Hence, adjunctive therapies have been attempted to enhance the outcomes of SRP in chronic periodontitis patients by specifically lowering the periodontal pathogens.

Laser-assisted periodontal therapy has been acknowledged as an adjunctive therapeutic arm due to its effective bactericidal effects and physical properties. The use of diode lasers (wavelengths range from 800 to 980 nm) has been widely accepted in dental armamentarium because of its simplicity of use and cost effectiveness. 4 Evidently, it holds some benefits with regard to periodontal therapy, that is, well absorbed by melanin, hemoglobin, and chromophores. 5 Also, scientific evidence has exhibited its ability to control periopathogenic bacteria, decrease systemic drug resistance, and undesirable effects on the healthy periodontal tissue. 6 In a systematic review authored by Qadri et al, 7 their observations rationalize that mechanical periodontal instrumentation followed by diode laser application led to the elimination of pocket epithelium compared to conventional SRP.

Currently, the annotations presented by the American Academy of Periodontology's Best Evidence Consensus, advocates that the available data is inadequate to ascertain if lasers when used an adjunct or alone can provide similar benefits as traditional periodontal therapy. Hence, more randomized controlled studies are warranted to elucidate their actual effects in comparison with SRP. 8

Concurrent to the benefits of adjunctive therapy, an oral tissue desiccant agent, HybenX (EPIEN Medical, Inc., Saint Paul, Minnesota, United States), with a distinctive technology have been marketed and used as a nonsurgical source of periodontal therapy along with SRP. Its ability to express high affinity for water permits denaturation and further desiccation of the formed biofilm matrix. As a result, the matrix contracts to detach it from the tooth surface. 9 HybenX has also been used in treating oral lesions like recurrent aphthous stomatitis and periodontitis. 10 11 Hence, this particular property of the topical decontaminant was utilized for further research to see if has an effective adjunct in the elimination of plaque biofilm.

Furthermore, infective status within the pocket environment can be studied by estimating cytokine profile, which are pivotal modulators of acute and chronic inflammatory reactions. During periodontal tissue destruction, initiated by specific bacteria, the cytokine network plays a crucial role on the recruitment of specific immunocytes, control of pathobionts, and release of cytokines such as interleukin (IL)-1, IL-6, IL-8, IL-10, IL-12, and tumor necrosis factor α (TNF-α). 12 13 Additionally, specific positive correlation between IL-1β and TNF-α in chronic periodontitis patients have also been reported. 14

Studies have evaluated the effects of HybenX and SRP as an adjunct for the treatment of chronic periodontitis. 15 16

However, our literature search found no studies which compared the *in vivo* effects of laser and the oral desiccant (HybenX) for the management of periodontal disease. Based on the views summarized above and the available evidence, the purpose of this present study was to evaluate the effect of SRP in combination with oral desiccant (HybenX) or laser irradiation on the clinical parameters and inflammatory mediators levels compared to SRP alone for the treatment of chronic periodontitis.

The null hypothesis was at the third-month follow-up, there were no variations between SRP plus HybenX or laser and SRP alone.

## Materials and Methods

**Fig. 1:**
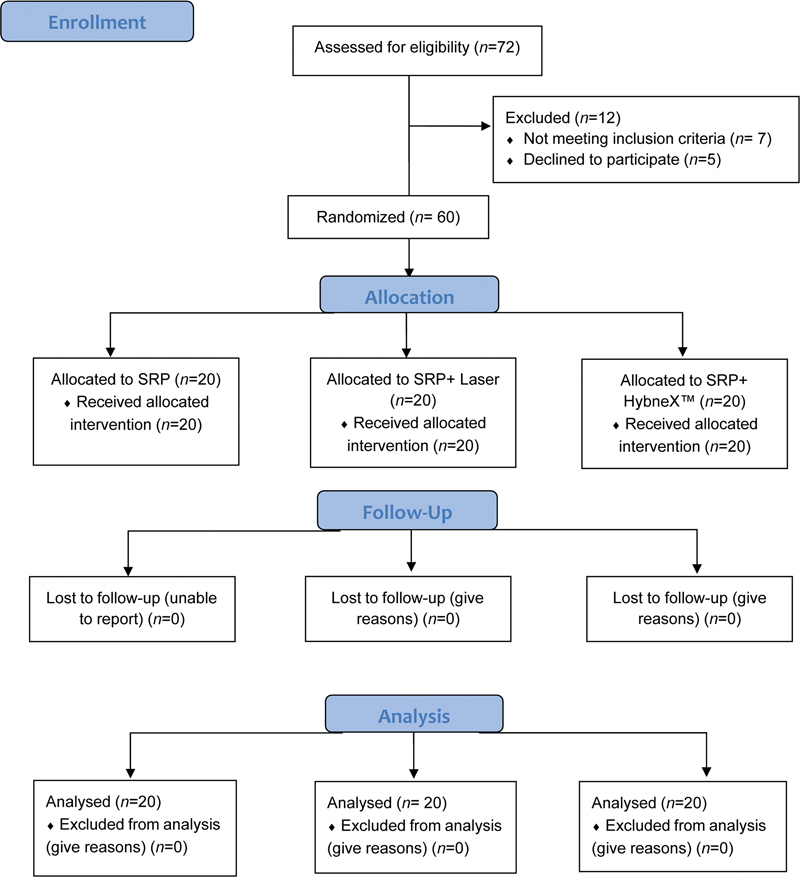
Study layout based on the Consolidated Standards of Reporting Trials (CONSORT).

This single-centered prospective randomized controlled three-arm parallel experimental clinical study included chronic periodontitis patients who visited the outpatient unit in the department of periodontology. The protocol was reviewed and approved by the Kasturba Medical College and Kasturba Hospital Institutional Review Board that was conducted in accordance with the Helsinki Declaration of 1975 further revised in 2013 and prospectively registered in the Clinical Trial Registry - India (CTRI/2019/01/016964). The clinical trial followed the CONSORT guidelines 2010 ( Fig. 1 ). 17

### Case Selection

Systemically healthy adults (age group 30–55 years) who met the inclusion criteria (1) patients with localized moderate to severe form of periodontitis with pocket probing depth (PPD) ≥ 6 mm and clinical attachment level (CAL) ≥ 3 mm (as per the 2017 World Workshop on the Classification of Periodontal and Peri-implant Diseases and Conditions) 18 and (2) presence of minimum of 20 functional natural teeth were recruited for this study. The exclusion criteria involved participants (1) allergic to sulfonated compounds, (2) history of systemic diseases, (3) use of antibiotics/anti-inflammatory medications for the last 3 months, (4) pregnant and lactating women, and (5) consumption of alcohol or smoking.

### Study Design

The initial examination was carried out by the coinvestigator (J.V.) in which total of 72 patients were screened and only those who met the inclusion criteria were included in the present study. Hence, only 60 patients matched the enrolment criteria. Based on enrolment, a block randomization was performed by using random sequence generation table. A block size of 6 samples and an allocation ratio of 1:1 was considered. Allocation concealment to the principle investigator (A.R.) was achieved by using sealed coded opaque envelopes for treatment allocation. The envelope was opened just prior to commencement of procedure. An investigator who was not engaged in the collection, recording, or processing of data assigned the indicated sealed envelopes before each treatment.

The principle investigator (A.R.) documented a detailed case history for all the study participants on a standard pro forma. An informed consent was taken from all the qualified patients prior to the commencement of the clinical trial. Further, baseline measurements of clinical parameters were recorded: plaque index (PI) (Silness and Loe, 1964), gingival index (GI) (Loe and Silness, 1963), 19 bleeding on probing (BOP), 20 PPD, and CAL. Gingival crevicular fluid (GCF) was procured for analysis of baseline inflammatory mediators.

Further, the study participants were randomly divided into three groups and received one of the therapeutic modalities described below:

Group 1: SRP with saline irrigation which served as control group.
Group 2: SRP with soft tissue laser irradiation using fiber size of 300 µm was performed in localized periodontal pocket at a power of 0.8 Watts, energy levels at 0.80 J/S. The mode of delivery was a continuous pulse.
Group 3: SRP with additional use of HybenX gel which was dispensed in a syringe. The needle was inserted into the deepest point of the pocket into which the gel was deposited. The time required for the session was 30 to 60 seconds and finally the diseased sulcus was irrigated with saline.

*Post-treatment instructions* : All the patients were trained to follow the modified Bass tooth brushing technique with a medium to soft toothbrush. They were specifically informed to abstain from any other oral hygiene measures during the trial period. The recall was scheduled after 1 month where only PI and GI were recorded. At the end of third month, BOP, PPD, and CAL were also assessed. 21 22

### GCF Sampling

The inflammatory mediators were analyzed in the GCF samples. The diseased tooth site to be sampled was isolated with cotton rolls and lightly air dried. A paper strip (Periopaper, Oraflow, Smithtown, New York, United States) was gently placed for 30 seconds into the deepest point of the pocket until a tissue resistance was felt. The samples were eluted at 4°C overnight into 500 μL phosphate buffer saline. Further, the paper strips were centrifuged at 400 × *g* for 4 minutes, and then removed. The supernatants were stored at –80°C for the estimation of IL-1β and TNF-α levels. 23

### Immunologic Analysis

Enzyme-linked immunosorbent assay (ELISA) method using an equation with software was used to determine the GCF levels of IL-1β and TNF-α. Both these inflammatory mediators were estimated using human IL-1β and TNF- α ELISA Kit (Clementia Biotech) as per the manufacturer's instructions.

### Statistical Analysis

As per the power analysis, the statistical data were analyzed using SPSS software 20. The sample size was based on the primary outcome variables which were reduction in PPD and gain in CAL in all the three experimental groups. The secondary outcome included the changes in the inflammatory mediators (IL-1β and TNF-α) in the groups. To achieve 80% power and detect significant differences in the clinical parameters between the groups, a total of 16 patients per group were required. To protect from possible dropouts, the sample size was increased to 20 patients per group.

Normality determination was checked using the Shapiro–Wilk test. Since, the data was not normally distributed, nonparametric tests were applied. Friedman test was used to compare the variables of PI and GI among the study groups at different time intervals. The nonnormally distributed data were expressed as median (interquartile range). The comparison of scores of GI, PI, BOP, PPD, and CAL between the groups was analyzed with Kruskal–Wallis test. Wilcoxon sign rank test was used to compare the pairwise comparison of clinical parameters among the groups and three related variables (BOP, PPD, and CAL) from baseline to the third month. A one-way analysis of variance test was used to compare the inflammatory mediators at different time intervals and paired *t* -test was used to compare the inflammatory mediators in each study groups. Statistical significance level of *p* < 0.05 was considered significant.

## Results

The present study aimed to evaluate the reduction in periodontal pocket depth after the use of adjunctive mode of therapies post-SRP and further to compare if either laser therapy or HybenX provided better outcome. No participants dropped out of the trial and the response rate was 100% at all recall visits. The mean age of patients was 42.33 ± 6.18 years which comprised of 34 males and 26 females (SRP alone group: 13 males and 7 females, laser group: 9 males and 11 females, HybenX group: 11 males and 9 females). No adverse events were reported from the participants.

At baseline, clinical parameters were comparable across the test groups. All participants displayed fair levels of PI and GI scores at baseline which was upgraded to significantly good levels at the end of the third month time interval ( Table 1 ). But analysis of these parameters between the study groups did not exhibit statistical significance ( *p* > 0.05). The BOP was observed in all participants at baseline which significantly returned to normal gingival status at the end of the third month recall visit ( Table 1 ). On comparison between the study groups, no statistical significance was observed among the groups ( *p* > 0.05).

**Table 1:** Comparison of PI, GI, and BOP between different time intervals in each study groups

PI		*N*	Mean (SD)	Range	Median (Q1–Q3)	*p* -Value
SRP	Baseline	20	1.26 (0.49)	0.3–2	1.1 (0.9–1.85)	< 0.001 *
	One month	20	0.78 (0.40)	0–1.2	0.9 (0.58–1.08)	
	Third month	20	0.53 (0.39)	0–1	0.7 (0.05–0.9)	
Laser	Baseline	20	1.25 (0.35)	0.9–2	1.1 (1.03–1.4)	< 0.001 *
	One month	20	0.85 (0.16)	0.6–1.1	0.9 (0.7–1)	
	Third month	20	0.48 (0.29)	0–1	0.4 (0.3–0.68)	
HybenX	Baseline	20	1.30 (0.43)	0.9–2	1.2 (0.93–1.78)	< 0.001 *
	One month	20	0.97 (0.26)	0.4–1.4	1 (0.9–1.18)	
	Third month	20	0.58 (0.35)	0–1	0.7 (0.3–0.9)	
GI						
SRP	Baseline	20	1.09 (0.37)	0.4–2	1 (0.9–1.18)	< 0.001 *
	One month	20	0.72 (0.31)	0.3–1.1	0.8 (0.4–1)	
	Third month	20	0.38 (0.37)	0–1	0.25 (0.05–0.7)	
Laser	Baseline	20	1.01 (0.18)	0.7–1.3	1.1 (0.83–1.18)	< 0.001 *
	One month	20	0.61 (0.13)	0.4–0.9	0.6 (0.6–0.7)	
	Third month	20	0.37 (0.09)	0.3–0.7	0.4 (0.3–0.4)	
HybenX	Baseline	20	1.29 (0.51)	0.4–2	1.1 (1–2)	< 0.001 *
	One month	20	0.89 (0.47)	0.2–1.9	1 (0.4–1.08)	
	Third month	20	0.58 (0.38)	0–1.1	0.55 (0.3–0.98)	
BOP						
SRP	Baseline	20	1.85 (0.88)	0–3	2 (1–2.75)	< 0.001 *
	Third Month	20	0.75 (0.72)	0–2	1 (0–1)	
Laser	Baseline	20	2.00 (0.65)	1–3	2 (2–2)	< 0.001 *
	Third Month	20	0.55 (0.51)	0–1	1 (0–1)	
HybenX	Baseline	20	1.90 (0.72)	1–3	2 (1–2)	< 0.001 *
	Third Month	20	0.35 (0.49)	0–1	0 (0–1)	

Abbreviations: BOP, bleeding on probing; GI, gingival index; PI, plaque index; SD, standard deviation; SRP, scaling and root planing.

* Denotes *p* < 0.05 Statistically Significant.

### Primary Outcomes

*PPD and CAL*

The primary outcomes of the present trial were the PPD and CAL, which at baseline presented a mean of 7.10 ± 0.79, 6.95 ± 0.83, and 7.15 ± 0.81 in groups 1, 2, and 3. Following treatment, a statistically significant reduction (6.25 ± 0.72, 4.35 ± 0.93, 3.70 ± 0.66) was observed ( Table 2 ). On comparing these parameters between the study groups, a statistically significant difference was noted between SRP and HybenX, in which the HybenX group ( *p* < 0.001) was found to be more effective than SRP alone. Similarly, between the HybenX and laser study groups, the HybenX group showed a statistically significant improvement ( *p* = 0.02) ( Table 3 ).

**Table 2:** Comparison of PPD and CAL between different time interval in each study groups

PPD Treatment		N	Mean (SD)	Range	Median(Q1-Q3)	p-value
SRP	Baseline 3 ^rd^ Month	20 20	7.10 (0.79) 6.25 (0.72)	6–8 5–7	7(6.25 - 8) 6(6 - 7)	<0.001 a
Laser	Baseline 3 ^rd^ Month	20 20	6.95 (0.83) 4.35 (0.93)	6–8 3 - 6	7(6 - 8) 4(4 - 5)	<0.001 a
HybenX®	Baseline 3 ^rd^ Month	20 20	7.15 (0.81) 3.70 (0.66)	6–8 3 - 5	7(6.25 - 8) 4(3 - 4)	<0.001 a
CAL Treatment						
SRP	Baseline 3 ^rd^ Month	20 20	7.10 (0.79) 6.25 (0.72)	6–8 5–7	7(6.25 - 8) 6(6 - 7)	<0.001 a
Laser	Baseline 3 ^rd^ Month	20 20	6.95 (0.83) 4.35 (0.93)	6–8 3 - 6	7(6 - 8) 4(4 - 5)	<0.001 a
HybenX®	Baseline 3 ^rd^ Month	20 20	7.15 (0.81) 3.70 (0.66)	6–8 3 - 5	7(6.25 - 8) 4(3 - 4)	<0.001 a

Abbreviations: CAL, clinical attachment level; PPD, pocket probing depth; SD, standard deviation; SRP, scaling and root planing.

a p < 0.05 statistically significant, p > 0.05 nonsignificant.

**Table 3:** Comparison of PPD and CAL between study groups at each time interval

PPD	Treatment	N	Mean (SD)	Range	Median(Q1-Q3)	p-value
**Baseline**	**SRP**	20	7.10 (0.79)	6 - 8	7(6.25 - 8)	0.72(NS)
	**Laser**	20	6.95 (0.83)	6 - 8	7(6 - 8)	
	**HybenX®**	20	7.15 (0.81)	6 - 8	7(6.25 - 8)	
**3rd** **Month**	**SRP**	20	6.25 (0.72)	5 - 7	6(6 - 7)	<0.001 a
	**Laser**	20	4.35 (0.93)	3 - 6	4(4 - 5)	
	**HybenX®**	20	3.70 (0.66)	3 - 5	4(3 - 4)	
**CAL**						
**Baseline**	**SRP**	20	7.10 (0.79)	6 - 8	7(6.25 - 8)	0.72(NS)
	**Laser**	20	6.95 (0.83)	6 - 8	7(6 - 8)	
	**HybenX®**	20	7.15 (0.81)	6 - 8	7(6.25 - 8)	
**3rd** **Month**	**SRP**	20	6.25 (0.72)	5 - 7	6(6 - 7)	<0.001 a
	**Laser**	20	4.35 (0.93)	3 - 6	4(4 - 5)	
	**HybenX®**	20	3.70 (0.66)	3 - 5	4(3 - 4)	

Abbreviations: CAL, clinical attachment level; NS, nonsignificant; PPD, pocket probing depth SD, standard deviation; SRP, scaling and root planing.

a p < 0.05 statistically significant, p > 0.05 NS.

### Secondary Outcomes

*TNF-α and IL-1β*

**Fig. 2:**
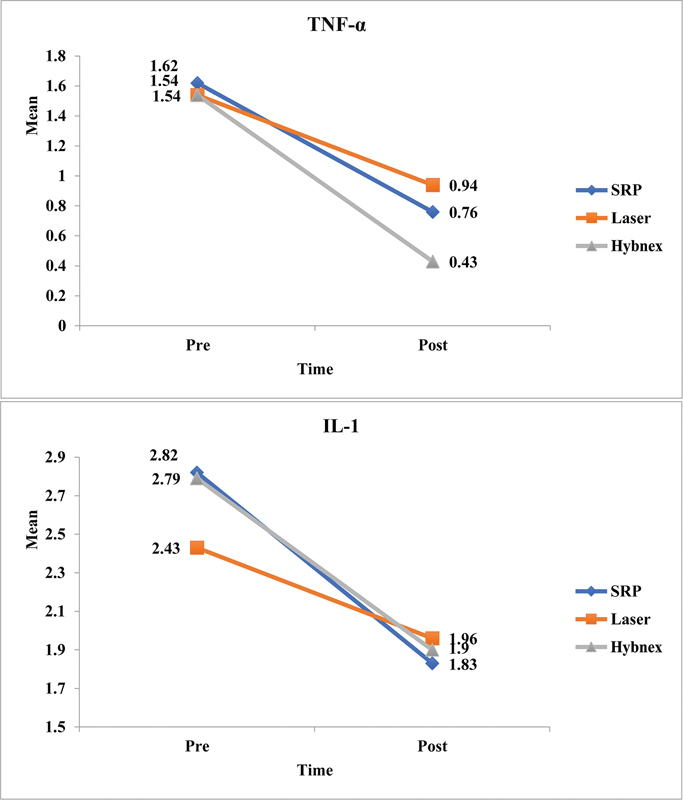
Comparison of tumor necrosis factor α (TNF-α) and interleukin (IL)-1 *β* between study groups.

The levels of TNF- α and IL-β were assessed in all the three study groups at the scheduled time intervals which decreased after the interventions ( *p* < 0.05). Intergroup comparison showed TNF-α levels in the HybenX group reduced significantly ( Fig. 2 ).

## Discussion

The current trial was an attempt to explore the therapeutic benefits of adjunctive procedures and its effect on diseased tooth prognosis. The notable observations at the third-month follow-up were statistically significant improvement in all the clinical parameters in the experimental groups. This beneficial effect could be attributed to the reinforced oral hygiene instructions informed to the trial participants. PI, GI, and BOP were assessed at regular study time intervals to discern the oral hygiene status maintained by the subjects during the study period and was found to be significantly reduced during all the clinical evaluation periods. 4 11 24 25

The primary outcome analyzed in this clinical trial was changes in PPD and gain in CAL after the use of diode laser and HybenX gel. These adjunctive therapies presented an overall reduction that occurred postoperatively (third month recall) compared to SRP alone.

Laser irradiation when used in permissible doses can be advantageous, further its combination with SRP has the potential to mend the diseased periodontal pockets compared to mechanical therapy alone.

Participants who received the diode laser therapy presented statistically significant results when compared to SRP alone at the end of third month recall period. These results were in agreement with two independent studies authored by Jia et al 26 and Pai et al. 27

Additional literature evidence, Yu et al concluded that diode laser as an adjunct to SRP exerted better clinical benefits and maybe proposed as an appropriate treatment for periodontitis at short-term time intervals. 28

Similarly, the use of chemo-desiccant revealed an overall enhanced improvement in all the clinical parameters compared to the SRP and laser groups.

In view of the results obtained in the present trial, there were clinical studies with contradictory reports. The use of diode laser did not demonstrate any superior improvement when compared to SRP alone. 29 Likewise, Lombardo et al did not observe any substantial change in the levels of PPD and CAL while using the topical desiccant (HybenX) along with ultrasonic debridement for chronic periodontitis therapy. 11

Proinflammatory cytokines contribute significantly to periodontal tissue damage, especially IL-1β and TNF-α. 30 31 32 In the present study, the levels of IL-1β and TNF-α reduced significantly in all the three experimental groups which were similar to the results demonstrated in previous published studies. 2 3 15 33

The present study revealed the beneficial role of HybenX gel as intrapocket medicament owing to its ability to enhance clinical parameters and decrease the amount of proinflammatory markers especially TNF-α, which resulted in statistically significant reduction. Scientific data suggests that host response causes greater periodontal tissue loss than bacterial damage. TNF-α was shown to have a vital function in triggering the innate host response and setting up the host defense against periodontal bacteria. 34

In context with the available literature and to the best of the authors' knowledge, this is the first clinical trial to demonstrate comparative evaluation of clinical and immunological parameters after administration of HybenX and diode laser along with SRP. The single application of laser or HybenX for beneficial pocket eradication outcome is still unclear. Also, the short duration (third month) follow-up may be among the main limitations of the present clinical trial.

## Conclusion

All the three experimental study groups resulted in amenable therapeutic development at the end of the third month evaluator period compared to baseline. One application of HybenX gel as an adjunct to SRP offered additional clinical and immunological benefits when compared to laser and SRP alone. Within the limitations of the study, the adjunctive use of HybenX gel with SRP did yield beneficial results.

## References

[1] Obeid P R D'Hoore W Bercy P Comparative clinical responses related to the use of various periodontal instrumentation J Clin Periodontol 2004 31 03 193 199 15016023 10.1111/j.0303-6979.2004.00467.x

[2] Tunkel J Heinecke A Flemmig T F A systematic review of efficacy of machine-driven and manual subgingival debridement in the treatment of chronic periodontitis J Clin Periodontol 2002 29 03 72 81, discussion 90–91 12787208 10.1034/j.1600-051x.29.s3.4.x

[3] Bartold P M Van Dyke T E Periodontitis: a host-mediated disruption of microbial homeostasis. Unlearning learned concepts Periodontol 2000 2013 62 01 203 217 23574467 10.1111/j.1600-0757.2012.00450.x PMC3692012

[4] Aoki A Sasaki K M Watanabe H Ishikawa I Lasers in nonsurgical periodontal therapy Periodontol 2000 2004 36 01 59 97 15330944 10.1111/j.1600-0757.2004.03679.x

[5] Raffetto N Lasers for initial periodontal therapy Dent Clin North Am 2004 48 04 923 936, vii 15464558 10.1016/j.cden.2004.05.007

[6] Raghavendra M Koregol A Bhola S Photodynamic therapy: a targeted therapy in periodontics Aust Dent J 2009 54 01 S102 S109 19737261 10.1111/j.1834-7819.2009.01148.x

[7] Qadri T Javed F Johannsen G Gustafsson A Role of diode lasers (800-980 nm) as adjuncts to scaling and root planing in the treatment of chronic periodontitis: a systematic review Photomed Laser Surg 2015 33 11 568 575 26436596 10.1089/pho.2015.3914

[8] Mills M P Rosen P S Chambrone L American Academy of Periodontology best evidence consensus statement on the efficacy of laser therapy used alone or as an adjunct to non-surgical and surgical treatment of periodontitis and peri-implant diseases J Periodontol 2018 89 07 737 742 29693260 10.1002/JPER.17-0356

[9] Antonelli A Giovannini L Baccani I Giuliani V Pace R Rossolini G M In vitro antimicrobial activity of the decontaminant HybenX® compared to chlorhexidine and sodium hypochlorite against common bacterial and yeast pathogens Antibiotics (Basel) 2019 8 04 188 31627304 10.3390/antibiotics8040188 PMC6963449

[10] Porter S R Al-Johani K Fedele S Moles D R Randomised controlled trial of the efficacy of HybenX in the symptomatic treatment of recurrent aphthous stomatitis Oral Dis 2009 15 02 155 161 19207485 10.1111/j.1601-0825.2008.01503.x

[11] Lombardo G Signoretto C Corrocher G A topical desiccant agent in association with ultrasonic debridement in the initial treatment of chronic periodontitis: a clinical and microbiological study New Microbiol 2015 38 03 393 407 26147153

[12] Pan W Wang Q Chen Q The cytokine network involved in the host immune response to periodontitis Int J Oral Sci 2019 11 03 30 31685798 10.1038/s41368-019-0064-z PMC6828663

[13] Genco R J Host responses in periodontal diseases: current concepts J Periodontol 1992 63 338 355 10.1902/jop.1992.63.4s.338 1573548

[14] Vahabi S Sattari M Taheraslani M Bagheban A A Correlation between interleukin- 1β, interleukin-6 and tumor necrosis factor-α and clinical parameters in chronic and aggressive periodontal disease J Adv Periodontol Implant Dent 2011 3 02 51 56

[15] Isola G Matarese G Williams R C The effects of a desiccant agent in the treatment of chronic periodontitis: a randomized, controlled clinical trial Clin Oral Investig 2018 22 02 791 800 10.1007/s00784-017-2154-7 28624914

[16] Khalil B Abou Sulaiman A Al Hajjar B The effects of adjunctive use of a desiccant agent in the treatment of stage III periodontitis (randomized controlled clinical trial) Saudi Dent J 2023 35 02 172 177 36942201 10.1016/j.sdentj.2023.01.001 PMC10024107

[17] CONSORT Group Schulz K F Altman D G Moher D CONSORT 2010 statement: updated guidelines for reporting parallel group randomized trials Ann Intern Med 2010 152 11 726 732 20335313 10.7326/0003-4819-152-11-201006010-00232

[18] Tonetti M S Greenwell H Kornman K S Staging and grading of periodontitis: framework and proposal of a new classification and case definition J Periodontol 2018 89 01 S159 S172 29926952 10.1002/JPER.18-0006

[19] Löe H The gingival index, the plaque index and the retention index systems J Periodontol 1967 38(6, 6 Part II):610 616 10.1902/jop.1967.38.6.610 5237684

[20] Mühlemann H R Son S Gingival sulcus bleeding–a leading symptom in initial gingivitis Helv Odontol Acta 1971 15 02 107 113 5315729

[21] Lowenguth R A Greenstein G Clinical and microbiological response to nonsurgical mechanical periodontal therapy Periodontol 2000 1995 9 01 14 22 9567975 10.1111/j.1600-0757.1995.tb00052.x

[22] Proye M Caton J Polson A Initial healing of periodontal pockets after a single episode of root planing monitored by controlled probing forces J Periodontol 1982 53 05 296 301 7045322 10.1902/jop.1982.53.5.296

[23] Guentsch A Kramesberger M Sroka A Pfister W Potempa J Eick S Comparison of gingival crevicular fluid sampling methods in patients with severe chronic periodontitis J Periodontol 2011 82 07 1051 1060 21235330 10.1902/jop.2011.100565 PMC3129431

[24] Konopka L Pietrzak A Brzezińska-Błaszczyk E Effect of scaling and root planing on interleukin-1β, interleukin-8 and MMP-8 levels in gingival crevicular fluid from chronic periodontitis patients J Periodontal Res 2012 47 06 681 688 22510045 10.1111/j.1600-0765.2012.01480.x

[25] Apatzidou D A Kinane D F Quadrant root planing versus same-day full-mouth root planing. I. Clinical findings J Clin Periodontol 2004 31 02 132 140 15016039 10.1111/j.0303-6979.2004.00461.x

[26] Jia L Jia J Xie M Clinical attachment level gain of lasers in scaling and root planing of chronic periodontitis: a network meta-analysis of randomized controlled clinical trials Lasers Med Sci 2020 35 02 473 485 31691054 10.1007/s10103-019-02875-5

[27] Pai B SJ Krishnan N R Walveker A Comparative evaluation of sclerostin levels in gingival crevicular fluid in the treatment of chronic periodontitis patients using diode laser as an adjunct to scaling and root planing: a clinico-biochemical study Contemp Clin Dent 2021 12 03 276 281 34759685 10.4103/ccd.ccd_19_20 PMC8525806

[28] Yu S Zhao X Zhang Y Liu Y Li A Pei D Clinical effectiveness of adjunctive diode laser on scaling and root planing in the treatment of periodontitis: is there an optimal combination of usage mode and application regimen? A systematic review and meta-analysis Lasers Med Sci 2022 37 02 759 769 34536183 10.1007/s10103-021-03412-z

[29] Hatipoğlu M Aytekin Z Daltaban Ö Felek R Firat M Z Üstün K The effect of diode laser as an adjunct to periodontal treatment on clinical periodontal parameters and halitosis: a randomized controlled clinical trial Cumhur Dent J 2017 20 03 152 160

[30] Belibasakis G N Bostanci N The RANKL-OPG system in clinical periodontology J Clin Periodontol 2012 39 03 239 248 22092994 10.1111/j.1600-051X.2011.01810.x

[31] Hanna R Dalvi S Amaroli A De Angelis N Benedicenti S Effects of photobiomodulation on bone defects grafted with bone substitutes: a systematic review of in vivo animal studies J Biophotonics 2021 14 01 e202000267 32857463 10.1002/jbio.202000267

[32] Mastromatteo-Alberga P Cárdenas L AE Correnti M Cytokines and MMPs levels in gingival crevicular fluid from patients with chronic periodontitis before and after non-surgical periodontal therapy J Oral Res. 2018 7 03 98 101

[33] Pesevska S Nakova M Gjorgoski I Effect of laser on TNF-alpha expression in inflamed human gingival tissue Lasers Med Sci 2012 27 02 377 381 21380536 10.1007/s10103-011-0898-x

[34] Graves D T Cochran D The contribution of interleukin-1 and tumor necrosis factor to periodontal tissue destruction J Periodontol 2003 74 03 391 401 12710761 10.1902/jop.2003.74.3.391

